# EPAT: a user-friendly MATLAB toolbox for EEG/ERP data processing and analysis

**DOI:** 10.3389/fninf.2024.1384250

**Published:** 2024-05-15

**Authors:** Jianwei Shi, Xun Gong, Ziang Song, Wenkai Xie, Yanfeng Yang, Xiangjie Sun, Penghu Wei, Changming Wang, Guoguang Zhao

**Affiliations:** ^1^Department of Neurosurgery, Xuanwu Hospital, Capital Medical University, Beijing, China; ^2^China International Neuroscience Institute, Beijing, China; ^3^School of Psychology and Mental Health, North China University of Science and Technology, Tangshan, China

**Keywords:** data processing, electrophysiology, electroencephalography, event-related potential, open source, user-friendly, MATLAB, toolbox

## Abstract

**Background:**

At the intersection of neural monitoring and decoding, event-related potential (ERP) based on electroencephalography (EEG) has opened a window into intrinsic brain function. The stability of ERP makes it frequently employed in the field of neuroscience. However, project-specific custom code, tracking of user-defined parameters, and the large diversity of commercial tools have limited clinical application.

**Methods:**

We introduce an open-source, user-friendly, and reproducible MATLAB toolbox named EPAT that includes a variety of algorithms for EEG data preprocessing. It provides EEGLAB-based template pipelines for advanced multi-processing of EEG, magnetoencephalography, and polysomnogram data. Participants evaluated EEGLAB and EPAT across 14 indicators, with satisfaction ratings analyzed using the Wilcoxon signed-rank test or paired t-test based on distribution normality.

**Results:**

EPAT eases EEG signal browsing and preprocessing, EEG power spectrum analysis, independent component analysis, time-frequency analysis, ERP waveform drawing, and topological analysis of scalp voltage. A user-friendly graphical user interface allows clinicians and researchers with no programming background to use EPAT.

**Conclusion:**

This article describes the architecture, functionalities, and workflow of the toolbox. The release of EPAT will help advance EEG methodology and its application to clinical translational studies.

## Introduction

Electroencephalography (EEG) is a cost-efficient technique with excellent temporal resolution and a non-invasive direct measure of neuronal activity, which reflects the electric fields that arise predominantly due to the synchronous activity of post-synaptic potentials at apical dendrites on the brain’s cortical surface. The wide application of electrophysiology, in particular EEG, has led to an explosion in knowledge about brain function and dysfunction implicated in psychophysiology, cognitive science, functional neurosurgery, and brain-computer interfacing. At the same time, its use continues to expand in fields such as neuroergonomics (Feyissa and Tatum, 2019; Beniczky and Schomer, 2020; Wei et al., 2022). Event-related potential (ERP) is a series of positive and negative waves associated with stimuli extracted from EEG signals by means of signal averaging (Hajcak et al., 2010, 2019). It corresponds to specific neural activities, such as perception, cognition, and emotion (Hajcak et al., 2019). ERP has been widely employed for early cognitive evaluation, differential diagnosis, quantitative assessment, prognostic intervention, and curative impact prediction in a variety of brain functioning disorders.

Over the past decades, a variety of commercial EEG/ERP systems have been developed for clinical and basic research, enriching the toolbox available to neuroscientists and clinicians. Among these, tools such as EMEGS, EEGLAB, Brainstorm, SPM, and Fieldtrip have significantly contributed to EEG/ERP data analysis (Delorme and Makeig, 2004; Oostenveld et al., 2011; Peyk et al., 2011; Tadel et al., 2019). Here, our MATLAB toolbox EPAT (EEG/ERP Process & Analysis Tool) presents itself as a valuable addition, designed to address specific challenges in EEG/ERP analysis. One crucial challenge is the crisis of EEG/ERP workflow reproducibility, which occurs because most of the processing is performed using in-house or custom tools and multiple heterogeneous software packages (Gruning et al., 2018). Furthermore, long continuous EEG recordings are contaminated by a range of artifacts, including those induced by experimental equipment such as monitor flickering, line noise, and poor electrode contact, in addition to common issues like electrode displacement, eye blinks, and muscular movements. These unwanted artifacts can obstruct the measurement of electrophysiological correlates of neural activation (Clayson et al., 2019). In addition, researchers typically have high degrees of freedom in terms of using different filters and criteria for artifact removal before conducting the actual analysis. Multiple datasets may also be collected and combined, including electromyography (EMG) and functional magnetic resonance imaging (fMRI). Indeed, the analysis of EEG data and its clinical implications are crucial for the diagnostic and therapeutic work of neurologists and neurosurgeons. However, many busy clinicians lack a solid coding foundation and the ability to combine datasets from various data sources (Pernet et al., 2020).

Here, we present a free and open-source MATLAB toolbox called EPAT that allows for efficient, and multiple processing of EEG/ERP (as well as electromyography, magnetoencephalography, and polysomnogram) analysis pipelines. EPAT can gather a sizable amount of EEP/ERP data in accordance with unified standard parameters and execute batch processing, which reduces the possibility of errors in single data. To ensure subsequent data verification and correction, EPAT can also define various parameters in batches and save process parameters throughout data processing. In EPAT, bad trials identified and labeled by researchers or clinicians based on criteria such as signal anomalies or visual inspection—along with data from bad electrodes and abnormal independent component analysis (ICA) components, are cataloged. This cataloged data, representing segments of EEG/ERP recordings with significant artifacts or noise, serves as a training dataset for artificial intelligence (AI) models within EPAT. This AI-driven approach aims to enhance the automated detection and exclusion of such compromised data in future analyses. Additionally, with a user-friendly graphical user interface (GUI), EPAT provides novice as well as expert users with an advanced multivariate EEG/ERP analysis technique. This technical report aims to give a brief introduction to its basic functionality and a walkthrough of the procedure for running the software. We finally presented a discussion of the potential applications of the pipeline and compared EPAT with other toolkits.

## Methods

### MATLAB programming environment

EPAT toolbox runs under the MATLAB programming environment. MATLAB (The MathWorks, Inc.) is a multi-purpose computer and programming language, widely used in scientific computing and engineering fields, that provides a user-friendly interactive interface and a rich function library, allowing users to quickly develop and deploy various complex algorithms for tasks such as mathematical modeling, algorithm development, data visualization, and data analysis. All mathematical functions can be used in add-on toolboxes, with the possibility of obtaining toolboxes at a cost from MathWorks. Additionally, many individual scientists and engineers also provide toolboxes (such as EPAT) for free. MATLAB is compatible with all major operating systems, thus enabling users to easily run toolboxes on various platforms.

### Integration with EEGLAB toolbox

EPAT toolbox is built upon EEGLAB, a widely used MATLAB toolbox for processing EEG data (He et al., 2011). EPAT provides a modular and interactive GUI based on EEGLAB. EEGLAB functions are used in EPAT’s processing steps for EEG signal browsing, EEG signal preprocessing (including interpolation, segment removal, etc.), and ERP graph plotting. In addition to integrating these features, EPAT also provides several unique functionalities, including event correction, event classification, and batch processing standardization. The GUI aims to guide novice users in quickly mastering EEG signal processing and supports the integration of alternative or additional processing modules for more advanced users.

### Algorithms

EPAT includes a variety of algorithms for EEG data preprocessing. For example, with a single-channel signal, multi-dimensional analysis can be conducted across time, frequency, and time-frequency domains. This includes straightforward numerical computations such as mean value and latency calculations, as well as advanced signal characteristic analyses like fast Fourier transform (FFT) and inter-trial phase coherence (ITPC). Furthermore, for multi-channel signals, the EPAT toolbox offers tools to determine channel connectivity. It provides several algorithms, including phase locking value (PLV), phase lag index (PLI), and phase synchronization index (PSI), to assess synchronization across multiple channels. Key steps instrumental in enhancing data quality include filtering, removing artifacts, and re-referencing, an essential process we use and integrate following the guidelines outlined in the Prep paper (Bigdely-Shamlo et al., 2015). This re-referencing step, as recommended, helps in minimizing potential biases and improving the overall integrity of the EEG signal analysis. The filtering process can be implemented by using a variety of filtering methods and provides a variety of filter windows for selection, such as rectangular and Hamming windows. These can be used to realize a variety of filtering methods such as low-pass, high-pass, and notch filtering.

Principal component analysis (PCA) and ICA are used to remove artifacts (Buzzell et al., 2022). To extract characteristic information and remove artifacts, we used the dimensionality reduction of data, original EEG channels with correlated data are recombined into a new dataset of unrelated components that reflect the indicators of the original data. Most people working in cognitive neuroscience use m-values between 10 and 30, so we default to the median value of 20, which is provided by EEGLAB. Based on the analysis of these m principal components, artifacts caused by eye movements can be easily identified and removed.

**Figure 1:**
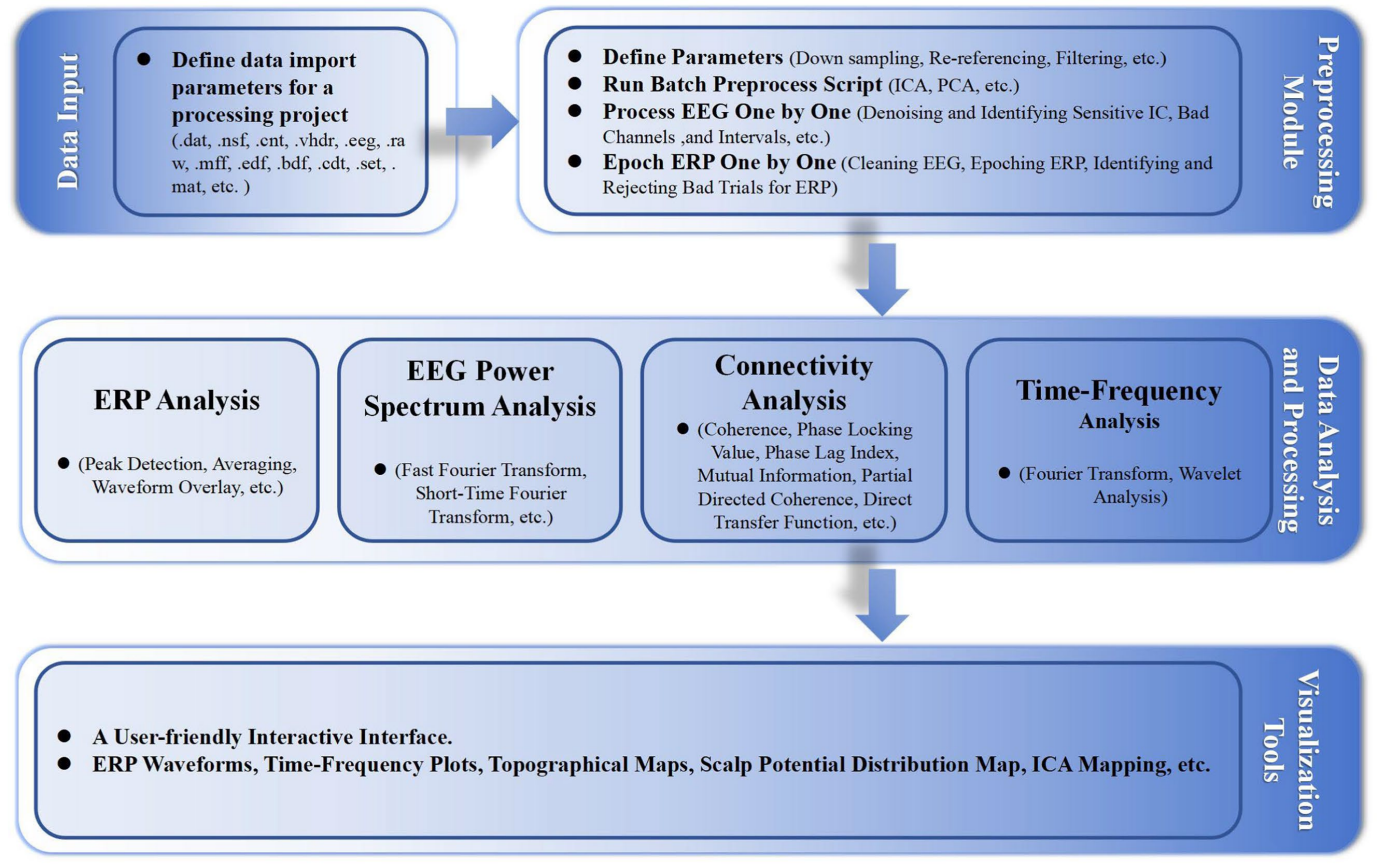
Illustrates the fundamental workflow and principal algorithms involved in the EPAT toolbox analysis. Comprising four main parts, it begins with the data input stage, supporting the import of various EEG file formats. The preprocessing module encompasses parameter settings, the execution of batch preprocessing scripts, and detailed processes like ‘Process EEG One by One’ and ‘Epoch ERP One by One’. The data analysis and processing phase includes ERP analysis, EEG power spectrum analysis, connectivity analysis, and time-frequency analysis. Finally, the toolbox offers a user-friendly interactive interface, alongside a suite of visualization tools for data representation. (EEG, electroencephalography; ERP, event-related potentials; ICA, independent component analysis; PCA, principal component analysis; ICs, independent components).

In addition, we developed some functionalities, including event correction and classification, and batch processing standardization, with highly customizable parameter settings. Figure 1 shows the basic workflow of the EPAT toolkit and the main algorithms involved in different steps. Moreover, EPAT not only provides flexible data export options but also ensures compatibility with commonly used statistical analysis software (such as SPSS) owing to its export format, thus facilitating the subsequent statistical analysis conducted by the user. The main code for EPAT can be obtained in the Supplementary file.

### Survey study

We invited several EEG researchers and clinical physicians from outside our center (Xuanwu Hospital, China International Neuroscience Institute) who have a certain level of knowledge in EEG analysis. They were asked to score their satisfaction with EEGLAB and EPAT from 14 different perspectives: Ease of installation and setup process, Intuitiveness of the user interface, Degree of user-friendliness for beginners, Comprehensiveness of manuals and user guides, Availability of training or tutorial resources, Compatibility with existing clinical workflows, Compatibility with other commonly used clinical software, Efficiency of batch processing features, Comprehensiveness of ERP analysis tools, Quality of time-frequency analysis outcomes, Speed of data processing and analysis tasks, Reliability of analysis results across different datasets, Convenience of data sharing after processing, and Willingness to recommend to others, with scores ranging from 1 to 5 (where 5 represents very satisfied). These scores were then compared. The Wilcoxon signed-rank test was applied if the score distributions were not assumed to be normal, or the paired *t*-test if the scores were normally distributed.

## Results

### Data structure and plotting

Just like EEGLAB/MATLAB and tools such as BioSig (Vidaurre et al., 2011), which have the capability to read EEG data formats provided by hardware manufacturers, EPAT also supports batch reading and saving of a wide range of EEG signal formats, including formats like .dat, .nsf, .cnt, and .set. This feature is designed to enhance user convenience, allowing researchers to seamlessly integrate data from various sources without needing to rely on multiple tools. It also ensures a consistent data processing workflow within EPAT, simplifying the steps required for comparative analysis across different EEG datasets and research projects. In addition, meta-parameters, analysis parameters, and processing results are saved in MATLAB format (i.e., data structure) in separate folders. Specifically, after completing each processing step, a MATLAB-compatible data file (*.mat file format) is generated, which facilitates users in reviewing and modifying the history of operations and results. These data files follow standardized naming conventions and are automatically or manually saved to the designated working folder, allowing for an overview of the current progress, and enabling quick transmission of critical files for comparison between different research projects.

### Statistical analysis

For users who need statistical analysis, such as conditional comparison, EPAT provides a dedicated GUI. In this interface, the significant difference of ERP components under different conditions is visualized to help users check the effect of preprocessing, to better understand and interpret the data. In addition, EPAT supports the export of the peak (or average amplitude) of a certain ERP component of each electrode and the time required to reach the peak (i.e., the latency period), which is convenient for users who want to perform subsequent T-tests, analysis of variance (ANOVA), linear mixed model (LMM), and other statistical analysis.

### Design concepts

The design concept and goal of EPAT are to provide an, efficient, easy-to-use, flexible, unified, and repeatable EEG data processing platform so that users can easily process and analyze complex EEG data. Its design concept mainly includes the following six aspects:

1. Standardization: EPAT employs a process-oriented pipeline, consistent with workflows recommended in EEG research, improving efficiency, and reducing errors (Gil Ávila et al., 2023). Tailored for clinical needs, its design focuses on resolving clinical issues rather than purely methodological advancements. EPAT also enables customization to meet different research needs, offering a balance between clinical workflows and basic research demands, facilitating future standardization of procedures.
2. Efficiency: EPAT’s efficiency is showcased through optimized algorithms, streamlined workflows, a well-designed codebase for execution and low overhead, high modularity for diverse EEG studies, a user-friendly interface easing the learning curve, and robust batch processing capabilities for large datasets, enhancing overall EEG research productivity.
3. Flexibility: The design of EPAT considers the needs of users with different expertise and in different specialties. It provides a variety of processing and analysis methods, allowing users to choose freely. For instance, novice users benefit from user-friendly default settings that simplify basic tasks such as preprocessing, making the software accessible to a wider audience. Conversely, for advanced users requiring detailed analyses, EPAT offers customizable options like diverse filters and event markers, catering to specific research needs and enabling intricate data manipulation.
4. Unification: EPAT employs an integrated approach, merging various data processing and analysis techniques into a unified platform. This platform offers users a tool for comprehensive analysis. EPAT’s unique data structure supports extensive analysis and allows for data export for additional analysis in other common software tools. The design of EPAT emphasizes tight integration between modules, each focusing on specific processing or analysis tasks while ensuring seamless data interfacing and transfer between them. This not only facilitates transitions between different analytical methods for the user but also simplifies and streamlines integration and data exchange with other software tools.
5. Repeatability: EPAT’s data structure is tailored for EEG data analysis, facilitating the repetition of crucial steps like removing bad segments and channels. This repeatability is important for manual intervention accuracy, bolsters the reliability of analysis outcomes. Users can retrace and modify previous processes within this framework, enhancing both the transparency and consistency of the workflow, and ensuring the dependability of results.

**Figure 2:**
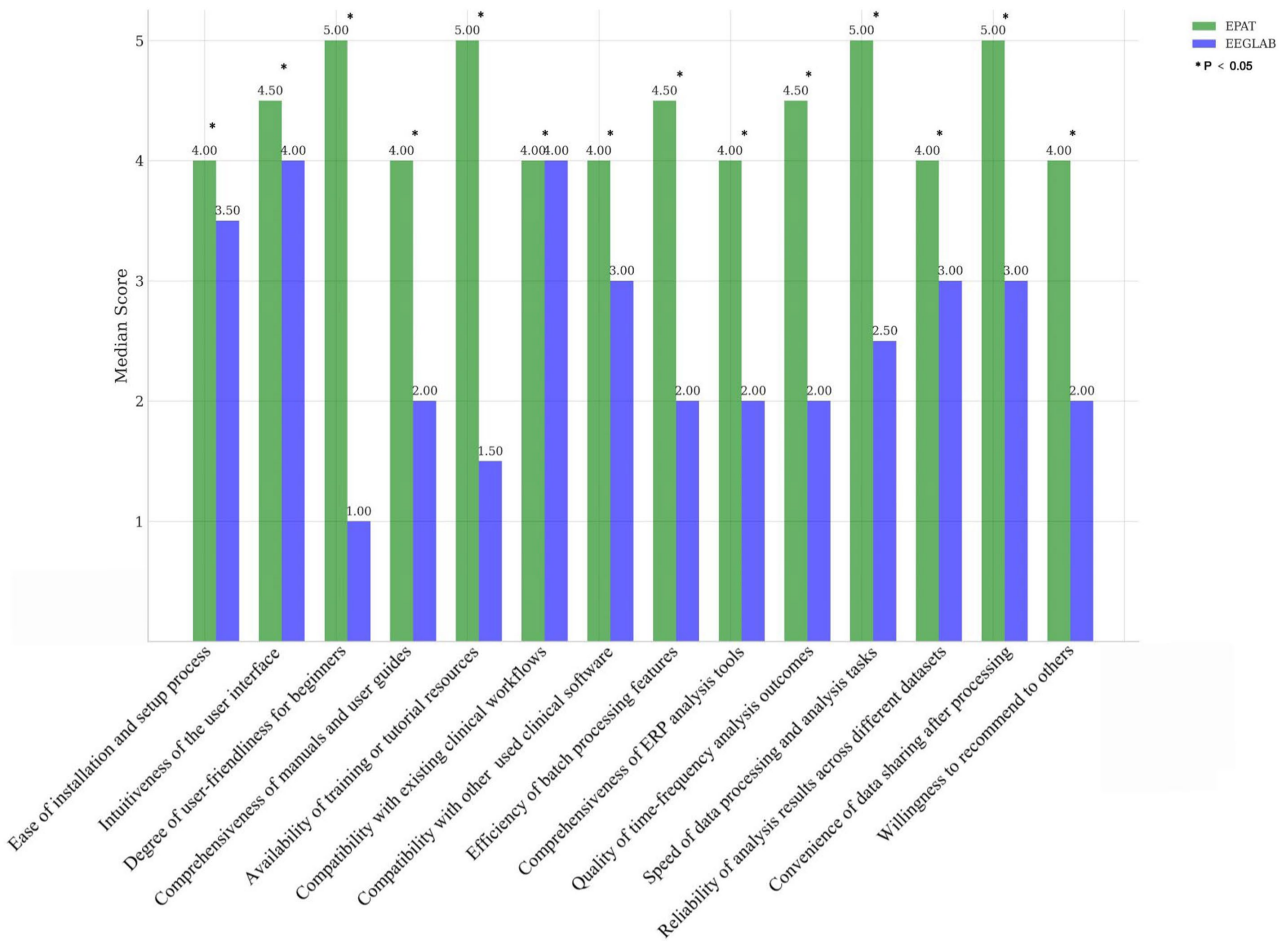
Comparison of EPAT and EEGLAB by 16 participants across 14 dimensions. * Indicates statistically significant differences.

Sixteen clinicians from the Neurosurgery and Neurology Departments were invited to participate in the scoring evaluation of EPAT and EEGLAB. The results, collected for 14 indicators and analyzed using the Wilcoxon rank-sum test, showed that EPAT was endorsed by the majority of participants (Figure 2, Survey Data).

### Features

EPAT, designed based on the concept described in the previous section, has the following characteristics: event correction and classification, highly customizable parameter settings, storage of data processing in the file format, ability to re-run ICA, flexible visualization tools, easily modifiable parameters, optimized storage scheme, flexible export scheme, and unique naming convention. We describe the key elements of these characteristics briefly here.

In the realm of scientific inquiry and clinical examinations, the process of event marking may encounter occasional disparities owing to a multitude of factors, including the skill of the operator, the quality of the equipment utilized, and the environmental conditions at hand. EPAT encompasses an event editing feature, allowing users not only to manually correct erroneous event markers on a case-by-case basis but also to rename events, adjust event latencies to account for known hardware/software delays, and group events into more meaningful categories (Bigdely-Shamlo et al., 2016). In instances where the event marking exhibits regularity, EPAT supports the batch import of correct event markers to replace erroneous ones, thereby supplanting the erroneous ones and thereby safeguarding the veracity of the triggered events. Moreover, during the phase of parameter definition, events can be judiciously categorized and pre-merged, thereby expediting subsequent comparisons and combinations of events, thereby bolstering the efficacy of data processing.

Parameter settings in EPAT are highly customizable and retrievable. Users can set global parameters for broad application across experiments, reducing the need for repetitive parameter adjustments and facilitating clinical EEG examinations. This helps users to comprehensively, quickly, and efficiently process large amounts of EEG data.

EPAT supports the integration of various EEG data formats and stores all traces and results generated during processing in the MATLAB-based. Mat format. This structured approach not only facilitates storage and retrieval of data for users but also simplifies integration with automated algorithms. Users can review the previous processing process by importing the corresponding file and adjusting as needed.

Due to inherent equipment noise and factors like electrical interference, raw EEG data invariably contain varying degrees of unavoidable noise. The presence of artifacts can significantly degrade the quality of EEG signals, necessitating the use of ICA to isolate specific components (Nakanishi and Miyakoshi, 2023). Experienced analysts can remove artifacts and noise based on their expertise, but for beginners, this task is not straightforward. EPAT offers a more intuitive visualization tool for ICA analysis results, clearly displaying the characteristics of different components, thus facilitating the process for novice users. However, despite such processing, noise and artifacts cannot be entirely eliminated and further analysis may be needed based on the actual conditions.

EPAT’s scaling function improves EEG data visualization, especially for high-density channel analysis. This allows for both detailed examination at the individual channel level and a comprehensive overview of the entire channel system, ensuring clarity and interpretability of data. In terms of temporal distribution, the zoom-in feature allows users to closely inspect specific events or short time frames, facilitating detailed analysis. Meanwhile, the zoom-out function provides an extensive view of longer EEG sequences, which is crucial for applications such as sleep stage analysis. These visualization tools are adept at aiding users of varying expertise in navigating and interpreting complex EEG data.

When users need to modify basic parameters, they often need to repeat ICA for subsequent analysis, which is very time-consuming for large amounts of data. In EPAT, the sensitivity of ICA decomposition to preprocessing parameters is considered. While ICA is highly sensitive to data preprocessing, not all parameter adjustments affect the ICA results. For instance, modifications to event combination parameters do not impact the completed ICA decomposition. Hence, EPAT allows users to readjust parameters that do not influence ICA components post data processing. Users simply need to update the relevant settings in the parameter file and then combine it with the existing ICA components for further analysis. This avoids the need to repeat the entire ICA process, enhancing efficiency in data processing. Moreover, this design offers flexibility in later stages of data processing, like after the removal of artifact segments, enabling users to make necessary parameter updates while preserving the integrity of the original processing. Thus, the easily modifiable parameters work with the ability to re-run ICA to achieve an efficient workflow.

Considering that the output content of time-frequency analysis takes up too much memory, EPAT utilizes a single-channel data storage scheme for saving time-frequency decomposition data, which is intended to minimize storage demands. During execution (RUN), although data from each channel is processed, we selectively retain only the data from channels of interest based on the research requirements. This means that while the raw data still contains comprehensive information across all channels, only critical channel data that is essential for subsequent analysis is preserved for storage. This approach effectively balances data integrity with storage efficiency, ensuring that even extensive time-frequency analyses can be conducted within the constraints of limited storage resources. The optimized storage scheme allows more data to be stored (the compression is 2x-4x).

EPAT supports flexible export of data according to user-defined latency frequency bands, electrode sites, and their combinations. For example, the data can be exported as Excel files. This facilitates users to flexibly select the parts of interest for statistical analysis and use the highly developed statistical software packages already available.

EPAT supports all data formats and organizes each subject’s data as a subset within the ‘data’ folder. This directory storage format is fundamentally aligned with the principles of the BIDS (Holdgraf et al., 2019; Pernet et al., 2019), simplifying the data organization process. In processing, EPAT automatically generates process files under the working path, including folders like ‘param’, ‘preprocess’, ‘result’, plus the ‘data’ folder, totaling four primary directories. This organization facilitates easy backup, review, and sharing. EPAT’s file naming reflects the creation time (including year, month, day, hour, minute, second), aiding in batch identification within the same process, especially in large datasets. It also prevents file overwriting, enhancing traceability and security in data processing. This naming strategy not only improves data management effectiveness but also aids in handling large data sets.

### Main functionality

As an EEG signal processing toolbox based on EEGLAB, EPAT can call almost all the functions of EEGLAB. On this basis, EPAT has optimized the single calls of EEGLAB according to the needs of users. The functions mainly include EEG data import, EEG data preprocessing, EEG data visualization, EEG data analysis, and EEG data export (see Table 1).

**Table 1:** Main functionality of EPAT.

Main functionality	Description
Import of EEG data	Supports batch import of electroencephalography (EEG) data in various formats, including DAT, NSF, CNT, VHDR, EEG, RAW, MFF, EDF, BDF, CDT, SET, and MAT (not only including the data formats that EEGLAB can import, but also supporting a variety of other data formats).
Preprocessing of EEG data	Includes electrode positioning, filtering, re-referencing, baseline correction, anti-artifact, condition combination, and other preprocessing methods. Also provides a variety of filters and filtering algorithms for users to choose. EPAT also supports a variety of denoise algorithms, such as independent component analysis (ICA), wavelet domain denoising, and principal component analysis. In particular, the preprocessing methods can realize batch setting and processing in the EPAT interactive graphical user interface (GUI) without coding.
Visualization of EEG data	Supports multiple visualization methods, such as event-related potential (ERP) waveforms, time-frequency maps, frequency domain power spectra, scalp potential distribution maps, and ICA decompositions. In particular, it supports multiple custom plotting options, such as channel selection, data trimming, and graph scaling.
Analysis of EEG data	Supports multiple methods of EEG data analysis, including ICA, ERP analysis, time-frequency analysis, and coherence analysis. It also supports analyzing statistical differences between graphical conditions, and users can further conduct statistical analysis by exporting data.
Export of EEG data	Supports naming and exporting various analysis result graphs such as ERP waveforms, time-frequency analysis graphs, and power spectrum graphs. Also supports exporting related data results. EPAT can also export information such as average reference waveform, event markers, and scalp electrode positions as MATLAB files.

### Advanced functionality

In addition to the aforementioned main functionality, EPAT can also provide many advanced functions for professional users. For example, when simultaneous scalp EEG and electromyogram (EMG) signals are available, EPAT can segment the two according to tasks, set references for them separately, and then conduct a coupling analysis of EEG and EMG. Within the data structure of EPAT, the files are compatible and the traces can be stored in the preprocessing process (compatible files can be matched or combined). This will facilitate the performance of more advanced analysis based on the existing data format and processing information, without having to go back for repeated processing. This capability helps users avoid wasting time. The traces of artificial map recognition can also be used as labels to accumulate data for AI, train AI, and contribute to big data analysis. EPAT also supports stereotactic EEG data analysis and results display without electrode positioning, and the analysis results can be directly imported into Brainstorm to draw a three-dimensional brain network connection map (Tadel et al., 2011; Cossu et al., 2017). No good display tool for bidirectional brain network analysis is available in the existing literature, and EPAT fills this gap.

In EPAT, we use the number of channels *n* as a denominator to calculate the average potential. While using *n + 1* is technically more accurate (Hyeonseok Kim et al., 2023), as it accounts for the influence of the original reference electrode, even when its actual value is zero, employing *n* is also commonly encountered in the literature. The drawbacks of using *n* instead of *n + 1* are only minor. The primary limitation is that using *n* instead of *n + 1* makes the data rank deficient by one (because ch1 + ch2 + ch2 + …chn = 0 is the definition of linear dependency), which leads the users to lose one independent component for no reason. Another subtle issue arises when users, albeit unlikely, need to re-reference the data multiple times. Utilizing *n* as the denominator contravenes the ‘no memory effect’ principle of re-referencing, precluding the possibility of returning to the original reference state. Despite these minor limitations, using *n* as a denominator is sometimes practiced probably because it is more intuitive (Hyeonseok Kim et al., 2023). EPAT provides a plugin called “review events” to help users browse through the event markers in EEG data and correct, modify, and update them. This is especially useful for response-related ERP, eye-movement–related ERP, and EEG-fMRI. Of course, users can also clear the data in that section (which will not affect further analysis) to help users mark the location for future use. EPAT also supports manual insertion of events, which can not only restore the authenticity of event data but also mark the occurrence of events, such as marking sleep stage types, sleep breathing events, and types of seizure attacks.

In clinical practice, various examination items may involve continuous data collection for multiple tasks. To handle data containing multiple tasks, EPAT provides a function called “segmentation” that allows users to split the data according to the task paradigm. The multiple tasks share the same ICA coefficients, and after segmentation, parameters can be set for each task for further analysis. In addition, the collection of clinical examination data involves large datasets and often long times, so EEG data is often processed in batches. For batch data with inherently different paradigms, EPAT provides a “Combine” function that allows users to integrate key files in batch data processing to obtain integrated data results. Regularly packaging data not only facilitates data sharing but also allows users to accumulate data based on a schedule (such as weekly, monthly, or quarterly). This more flexible data storage method also helps users retrieve and review relevant data as needed.

For data export, EPAT not only provides flexible export options but also ensures compatibility with commonly used statistical analysis software (such as SPSS) through its export format, which facilitates efficient statistical analysis by users.

### Usage of EPAT toolbox

EPAT provides clear visualization of the sequence and content of data processing. By setting curing parameters according to the inherent experimental paradigm, clinical EEG examination is facilitated. The installation of EPAT toolbox is accomplished in three steps, as follows: (1) Downloading the file “EPAT V4.9” (Supplementary file S1). (2) Running the MATLAB command “Set path,” selecting “Add with subfolders,” then EPAT_V4, and saving. Additional self-created total working folders may also be included by the user. (3) Entering “EPAT” into the MATLAB command window. Here, we will provide with a brief overview of its usage. We have prepared a demonstration video and test data so that readers can better understand the application of EPAT (Supplementary file S2). We also included an EPAT user manual with this article for the convenience of the readers (Supplementary file S3). You can also access related documents by visiting the initial official website of EPAT at https://www.epat-xwhosp.cn/.

**Figure 3:**
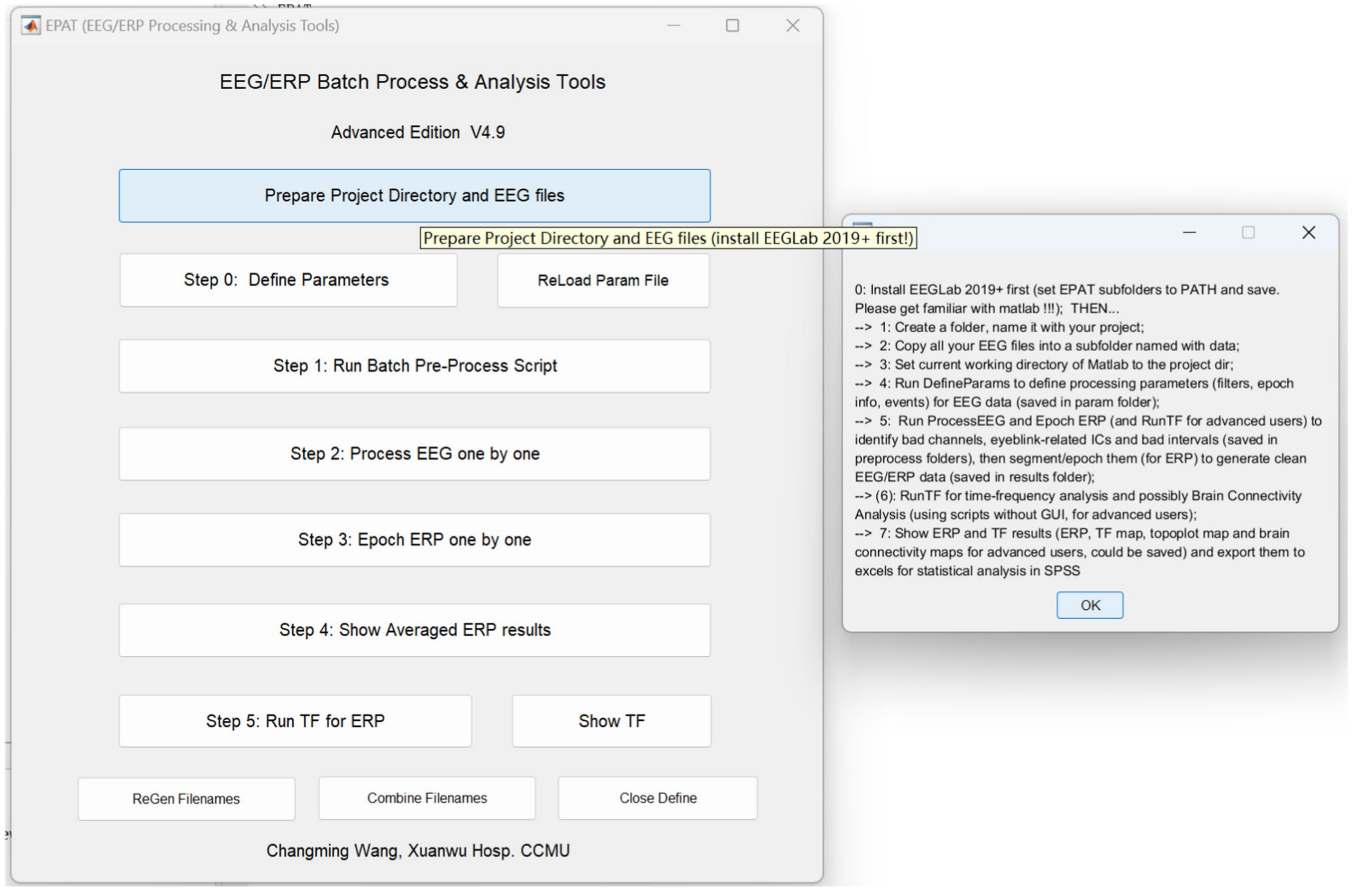
Schematic representation of the EPAT GUI and project directory and EEG file preparation. (GUI, graphical user interface; EEG, electroencephalography).

EPAT can analyze EEG data in a variety of forms and distinct data formats. After preparing the project directory and EEG files, the processing steps of EPAT toolbox (Version 4.9) are quite easy, consisting of the following steps (see Figure 3): Step 0. Define Parameters (in which ERP events and conditions parameters are defined); Step 1. Run Batch Preprocess Script (in which the user runs batch preprocess script for EEG from all subjects); Step 2. Process EEG One by One (in which the EEG data is reviewed and denoised, and sensitive ICs, bad channels, and intervals are identified); Step 3. Epoch ERP One by One (to clean EEG data and epoch ERP, and identify and reject bad trials for ERP); Step 4. Show Averaged ERP Results (in which averaged ERP between conditions from multiple subjects are displayed); and Step 5. Run TF for ERP (to complete the time-frequency analysis in batches for ERP). Here is a brief introduction.

### Step 0. Define parameters

**Figure 4:**
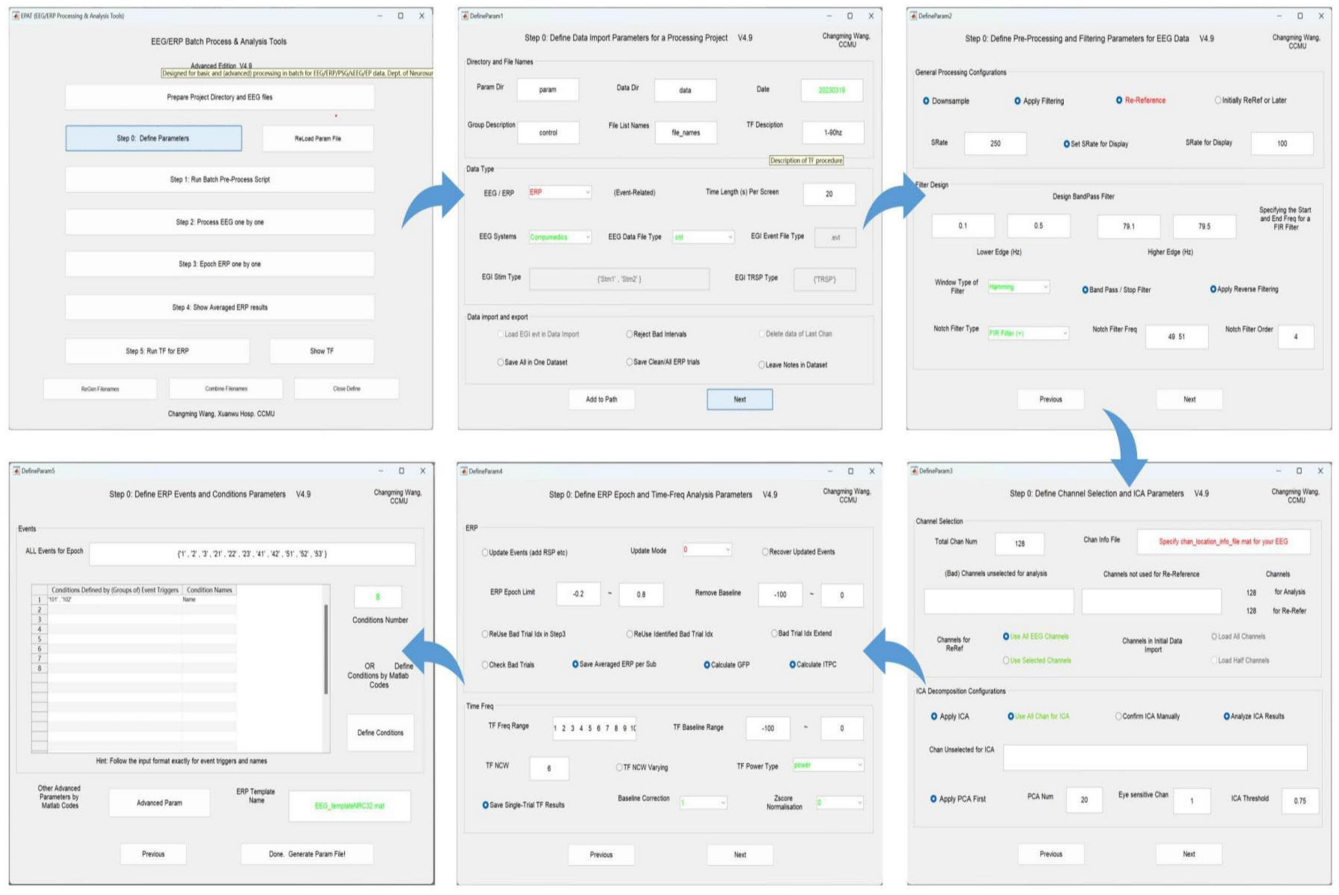
Defining parameters in EPAT. In general, five steps are required to complete the setting of all parameters. DefineParam1: Define data import parameters for a processing project. DefineParam2: Define preprocessing and filtering parameters for EEG data. DefineParam3: Define channel selection and ICA parameters. DefineParam4: Define ERP epoch and time-frequency analysis parameters. DefineParam5: Define ERP events and conditions parameters. After clicking “Next,” a separate new interface will pop up (the previous interface will not disappear). (ICA, independent component analysis; EEG, electroencephalography; ERP, event-related potential).

A total of five setting interfaces are included to complete the definition of parameters (see Figure 4). The initial data in the interface are default values, and the user should complete the settings and then click “Next.” In the DefineParam1 interface, the user selects the project file and describes the new file (the parameter file name defaults to “param”). Then the data file type and stimulus type are set, making sure the red-marked positions are selected; the green-marked positions are reminders for the operator to check. In DefineParam2, the downsampling of the original data is implemented to enhance processing efficiency, filtering is applied to eliminate noise from the original signal, and re-referencing is performed to mitigate the impact of the reference electrode itself. DefineParam3 is primarily utilized for selecting channels for data analysis, as well as configuring principal components analysis (PCA)/ICA decomposition. Users can define ERP epoch and time-frequency analysis parameters in DefineParam4. Finally, users select and fill in the event for epoch in DefineParam5 and click “Done.” Three new subfolders will be generated under the general folder. Helpful hints will appear as the mouse is moved to the button in each step.

### Step 1. Run batch preprocess script

**Figure 5:**
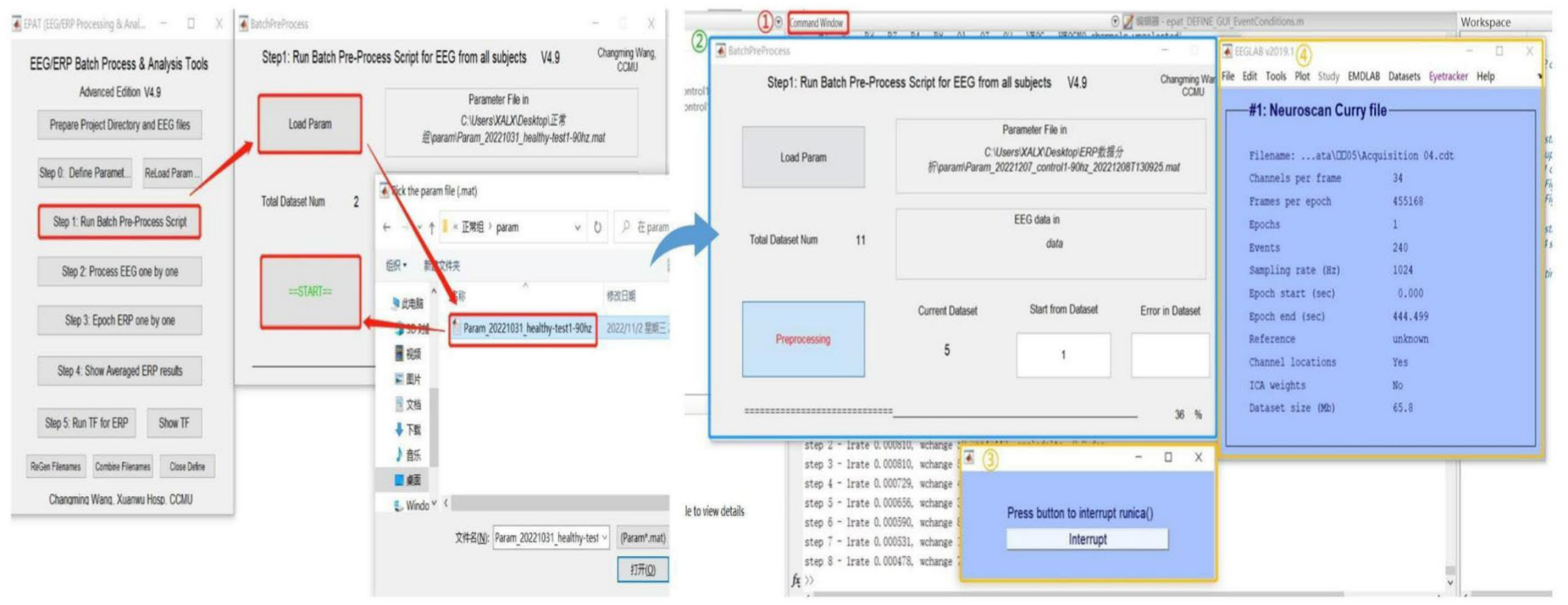
Running the batch preprocess script in EPAT. After clicking “Step 1 Run Batch Preprocess Script” and starting the analysis, four windows appear: 1. The MATLAB command window. 2. EPAT analysis progress. 3. The Pause window. 4. The EEGLAB information pop-up window.

After setting the parameters, users proceed to Step 1 for ICA or PCA analysis of batch data (see Figure 5). Clicking “Load Param” allows users to select the parameter files in the “param” folder, and then clicking “Start” begins the analysis. There will be four displays during this process: 1. The MATLAB command window, which will be running as usual. 2. EPAT analysis progress. 3. The Pause window (users are advised not to click “Interrupt” during analysis). 4. The EEGLAB information pop-up window.

### Step 2. Process EEG One by One

**Figure 6:**
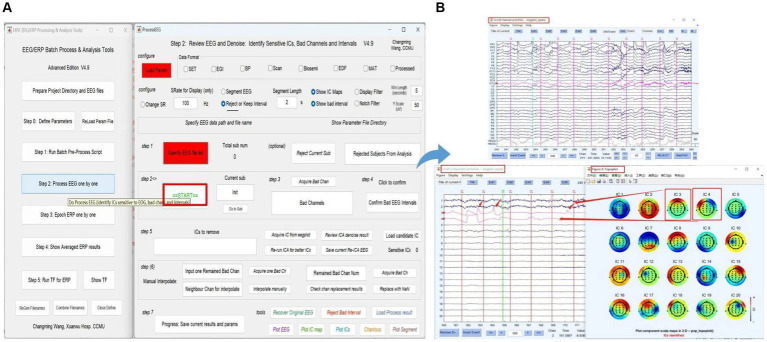
Processing EEG data in EPAT. The process EEG One by One step contains a total of seven steps. **(A)** General page layout. **(B)** The scroll channel activities, scroll component activities, and topoplot map obtained from the loaded data. (EEG, electroencephalography).

The next phase of using EPAT involves reviewing EEG data to denoise and identify sensitive ICs, bad channels, and intervals following the steps shown in Figure 6. After loading the parameter file (same as Step 1), the “Processed” option is ticked. The EEGLAB toolbox is opened using “Specify,” and then the user clicks “start” to display the scroll channel activities, scroll component activities, and topo plot map. Based on these figures, users can sequentially identify and remove bad channels and artifact components by following the instructions. The remaining data is then processed similarly until “The Last sub!” is prompted in Step 2, indicating that all processing is about to be completed. The results and parameters are saved to complete this step.

### Step 3. Epoch ERP One by One

**Figure 7:**
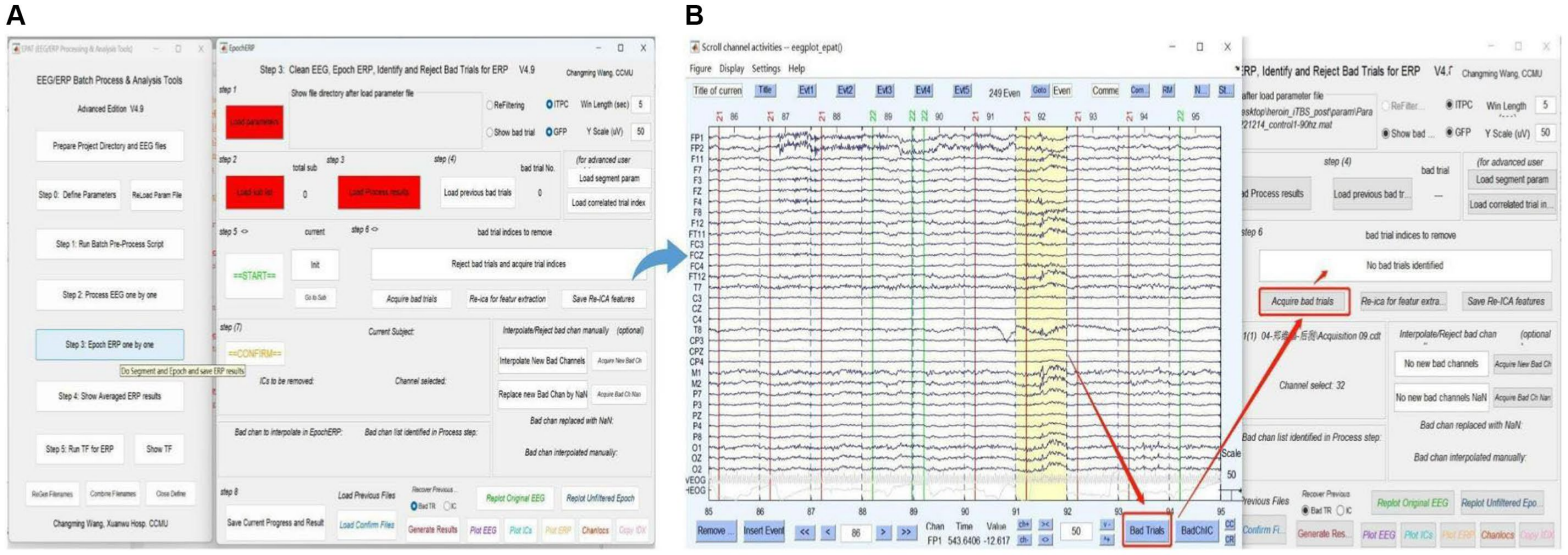
Processing ERP data in EPAT. The Epoch ERP One by One step contains a total of eight steps. **(A)** General page layout. **(B)** Analysis process after clicking “Start” in Step 3: First, users browse through each trial from the beginning, then click the bad trial with the left mouse button and mark it. After marking, users click “Bad trials” in the lower right corner, then return to the EpochERP interface and click “Acquire bad trials”; the corresponding bad trial number will appear in the upper space. (ERP, event-related potential).

Users clean EEG, epoch ERP, and identify and reject bad trials for ERP following Steps 1–8 in Figure 7. The commands “Load parameter,” “Load sub list,” and “Load Process Results” are used to import the data. After clicking “START,” users can select and mark the bad trials in scroll channel activities-eegplot. Then the EpochERP interface prompts users to “CONFIRM” bad trials. Users must wait for a while until the “CONFIRM” button turns from red to green. If no bad trials were identified in the data, users still need to click “CONFIRM”; otherwise, the data will be deleted by default. The remaining data is processed similarly until “The Last sub” is prompted.

### Step 4. Show averaged ERP results

**Figure 8:**
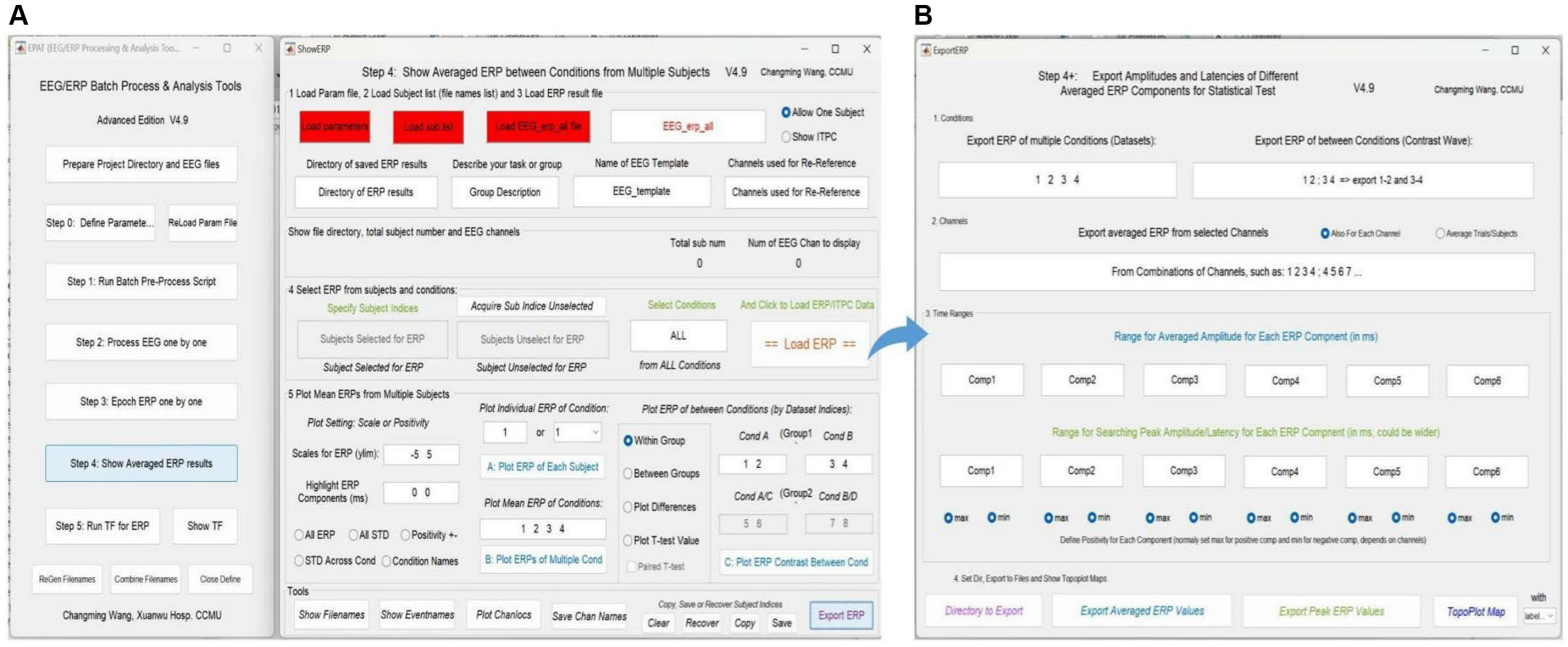
Averaged ERP results in EPAT. **(A)** There are five modules (1. Load parameter file; 2. Load subject list; 3. Load ERP result file; 4. Select ERP from subjects and conditions; and 5. Plot mean ERPs from multiple subjects) and a toolbar (show filenames, show event names, plot channel locations, and save channel names) in the Step 4 (Show Averaged ERP Results) interface. **(B)** Export amplitudes and latencies of different averaged ERP components (in the form of Excel) for statistical tests. (ERP, event-related potential).

Averaged ERP results between conditions from multiple subjects can be shown (see Figure 8). In addition, the test values for custom statistical analysis can be extracted. The Step 4 interface comprises five modules and a toolbar. Modules 1–3 are used to load the corresponding files, and allow the user to select “Allow One Subject” to show the amplitude of ERP and “Show ITPC” to show the inter-trial phase-coherence *g*. In Module 4, users can select ERP from subjects and conditions to load ERP/IPTC data. Module 5 is used to plot ERP from multiple subjects. Users can adjust scales for ERP (ylim, the range of the y-axis) and highlight ERP components (waveform in a certain time window), plot individual/mean ERP of one or more conditions, and plot ERP under different conditions within/between groups.

In the toolbar, filenames and event names can be displayed, channel locations can be plotted, and channel names can be saved. Subject indices can be cleared, recovered, copied, and saved (Generally, there is no need to use this feature, because EPAT will operate automatically). Clicking “Export ERP” causes a new operation interface to pop up. Following the prompts, users can extract the average and extreme values of ERP components in a certain time window according to the comparison between events (conditions) and the combination of electrodes for subsequent data statistics.

### Step 5. Run TF (time-frequency analysis) for ERP

**Figure 9:**
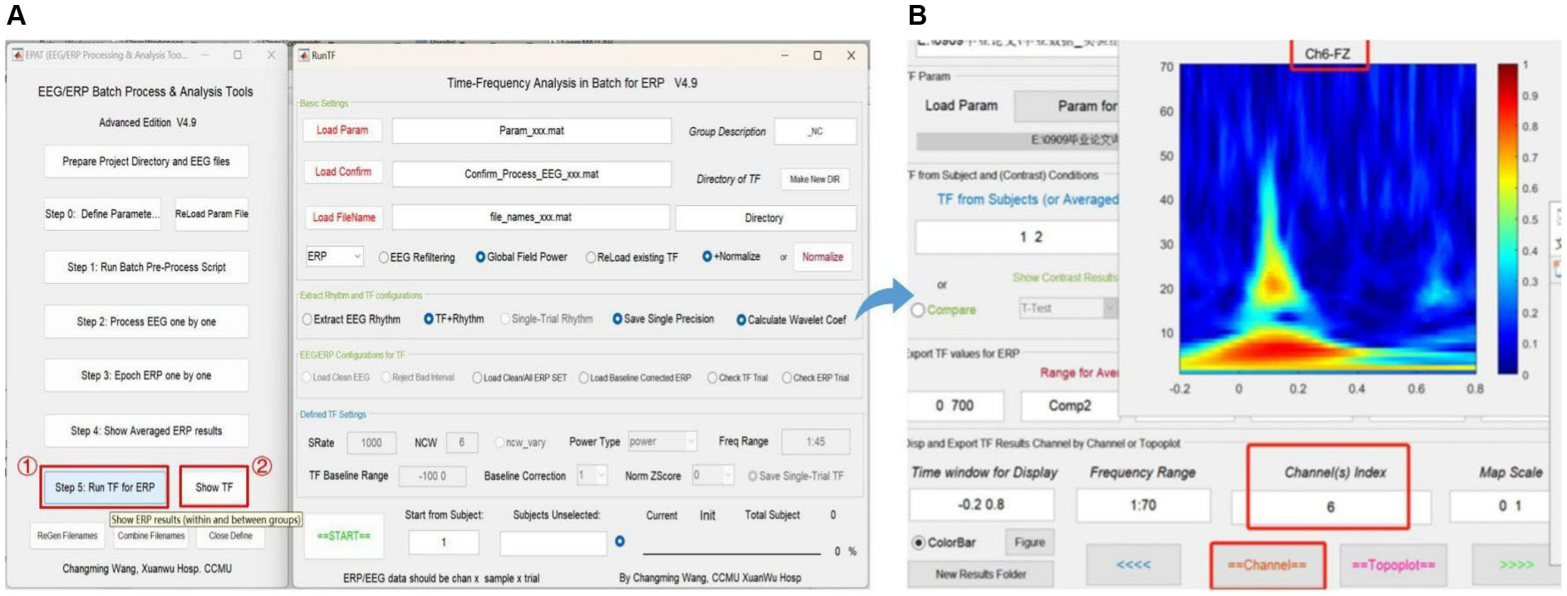
Time-frequency analysis in EPAT. Step 5 (Run TF for ERP) contains four modules. **(A)** General page layout: Module 1: Basic settings; Module 2: Extract rhythm and TF configurations; EEG/ERP configurations for TF; Module 3: Defined TF settings; and Module 4, which is used to track the procedure (at the bottom). **(B)** After clicking “Show TF” and entering the time window, frequency range, and channels of interest, EPAT will show and export TF results for ERP. (TF, time-frequency; ERP, event-related potential).

Step 5 is critical for TF analysis in batch data for ERP (see Figure 9). There are four modules in total. Module 1 (Basic settings) is used to import and load data. Settings in Modules 2 (Extract rhythm and TF configurations, EEG/ERP configurations for TF) and 3 (Defined TF settings) normally do not need to be altered. These first three modules are preset in Step 0. Module 4 is used to track the procedure. Clicking “Show TF” displays and exports ERP time-frequency results through another interface. If users want to see the TF map of a specific channel or the entire brain, they should check the function column entitled “Disp and Export TF Results by Channels or Topoplot.” To obtain exact result data of time-frequency analysis, the function column to focus on is “Export TF values for ERP,” and subsequently the data can be organized and statistical analysis executed in accordance with the specific research goal.

## Discussion

As a user-friendly, and powerful tool for processing EEG signals, EPAT is expected to play an important role in scientific research and clinical applications. One of the main reasons why EPAT’s various functions are currently implemented in the MATLAB environment is that EPAT is compatible with existing MATLAB-based EEG signal analysis tools, such as EEGLAB and BrainStorm (Delorme and Makeig, 2004; Tadel et al., 2011). In the future, EPAT will also be implemented and integrated with more neuroscience tools on platforms such as Python to facilitate users with different professional backgrounds.

No good display tool for bidirectional brain network analysis is available in the existing literature, and EPAT fills this gap (Delorme et al., 2011). EPAT provides tools for displaying bidirectional brain network connectivity, aiding users in visualizing the connections between brain regions to better understand the interactions within the brain. This includes three-dimensional brain network graphs and connectivity strength maps, allowing users to intuitively observe the connection patterns between different brain areas. Furthermore, EPAT offers algorithms and techniques for analyzing bidirectional brain network activity, helping users identify and quantify the functional connections and information transfer between different brain regions. This enables a deeper understanding of the dynamic properties of brain activity, such as functional connectivity strength, network centrality metrics, and spectral clustering algorithms for identifying functional brain network modules and subnetworks.

Our toolbox is built on the foundation of EEGLAB, a commonly used EEG data processing toolbox. We also considered Fieldtrip, another MATLAB-based EEG/MEG data processing toolbox (Delorme and Makeig, 2004; Oostenveld et al., 2011). However, using Fieldtrip functions appropriately requires a certain level of expertise and programming experience from the user. EPAT provides a comprehensive and streamlined standalone workflow GUI for EEG data processing, together with other toolboxes in the field to collectively enhance EEG analysis. Moreover, EPAT supports several file formats, facilitating the import of batches with different data types. For the data preprocessing stage, EPAT provides a variety of scripts and flexible visualization tools, including functions, such as re-run ICA and update trigger, that ensure the authenticity and effectiveness of the results. To facilitate users with different professional backgrounds and improve data processing efficiency, batch processing functions were modularized and incorporated into the visualization interface. Consequently, performing ICA and bad segment and/or channel removal on large batches of data does not require coding. Notably, EPAT supports multiple visualization methods, including ERP waveforms, time-frequency analysis graphs, frequency domain power spectra, scalp potential distribution maps, and ICA maps. EPAT also provides various custom plot options, such as channel selection, data cropping, and graph scaling. In terms of computational efficiency, we optimized several algorithms to obtain a competitive computational speed. A performance comparison of EPAT with other relevant toolboxes is presented in Table 2.

**Table 2:** Performance comparison with existing toolboxes.

Feature/Toolkit	EPAT	EEGLAB	FieldTrip
File format	Supports multiple data formats and can read them directly.	Supports EEG format only; alternatively, requires prior data format conversion.	Supports multiple data formats and can read them directly.
Data preprocessing	Supports scripting and flexible visualization tools, including intrinsic paradigms with fixed parameter settings (e.g., locating electrode points, re-referencing, filtering), trigger update, batch ICA, and batch bad segment(s) and/or channel(s) removal.	Provides a rich library of plugins and visualization tools, including basic preprocessing and filtering, and scripts for the removal of eye blink artifacts and spurious correlations.	Provides some preprocessing plugins and offers basic preprocessing functions (e.g., mean and trend removal, filtering).
Visualization	Provides flexible visualization tools, including ERP waveforms, time-frequency analysis, frequency domain power spectrum, scalp potential distribution map, and ICA mapping, with multiple customizable plotting options.	Provides an intuitive data visualization interface, supporting time series plots, power spectra, and topographic maps with limited customizable plotting options.	Provides an intuitive data visualization interface, supporting matrix plots, and topographic and source analysis maps with limited customizable plotting options.
User-friendliness	Provides a clear streamlined GUI interface for visual operations supporting direct batch processing.	Provides a basic GUI interface for visual operations. Batch processing requires MATLAB scripting.	Does not provide a GUI interface; all functions are implemented by coding.
Expandability	Provides a rich script library that can be extended with customized functions.	Provides a rich script library that can be extended with customized functions.	Provides a limited library of plugins that can be used to add customized functions.
Computational speed	Includes several optimized algorithms, resulting in a high computational speed.	Displays poor computational speed for large datasets.	Includes several optimized algorithms, resulting in a relatively high computational speed.
Learning curve	Does not require programming skills and presents a relatively smooth learning curve.	Requires basic MATLAB programming skills and presents a steep learning curve is steep.	Requires basic MATLAB and C++ programming skills and presents a steep learning curve.

However, EPAT presents some limitations concerning multi-signal data processing. In particular, EPAT can be mainly used for processing and analyzing EEG signals because it offers limited resources for the analysis of multimodal data and simultaneous processing of signals with different data types. In addition, EPAT is yet to support real-time signal processing and analysis. Although EPAT provides some basic statistical analysis functions, such as single-sample and paired-sample t-tests, it does not provide the tools for advanced statistical analyses, such as multiple comparisons, nonparametric statistical analysis, and mixed effects models. To perform these, other tools such as SPSS and R toolboxes should be used in combination with our toolbox. Our future research will aim at addressing these aspects. Moreover, we are currently collecting users’ feedback to further improve and optimize EPAT.

As more data processing methods are developed in the future, we will consider integrating these new solutions into the EPAT toolbox. With such advances, we will reasonably increase the number of GUI modules and visual features in EPAT to improve user experience. In the future, we will continue to improve and enhance the functionality, practicality, flexibility, and compatibility of EPAT to meet the growing scientific needs and technological advancements. Additionally, we will strive to establish more automated and intelligent EEG processing tools. EPAT can retain human annotation traces during the data processing process, which can accumulate materials for AI training, be applied to big data analysis, provide a basis for developing intelligent annotation, and further improve the speed and reliability of EPAT’s data analysis.

## Data availability statement

The datasets presented in this study can be found in online repositories. The names of the repository/repositories and accession number(s) can be found in the article/Supplementary material.

## Author contributions

JS: Writing – original draft, Writing – review & editing. XG: Writing – original draft, Writing – review & editing. WX: Methodology, Software, Writing – original draft. YY: Investigation, Methodology, Writing – review & editing. XS: Data curation, Formal analysis, Investigation, Writing – review & editing. PW: Investigation, Methodology, Software, Supervision, Validation, Writing – review & editing. CW: Investigation, Methodology, Software, Supervision, Writing – review & editing. GZ: Funding acquisition, Investigation, Software, Supervision, Validation, Writing – review & editing. ZS: Writing – original draft, Writing – review & editing.
